# Characterization of Genetic Mutations in Multi-Drug-Resistant Isolates of Mycobacterium tuberculosis Bacilli Conferring Resistance to a Second-Line Anti-tuberculosis Drug

**DOI:** 10.7759/cureus.40442

**Published:** 2023-06-14

**Authors:** Raj Kishor Sharma, Usha Kumari, Namrata Kumari, Rakesh Kumar

**Affiliations:** 1 Microbiology, Patna Medical College, Patna, IND; 2 Biochemistry, Indira Gandhi Institute of Medical Sciences, Patna, IND; 3 Microbiology, Indira Gandhi Institute of Medical Sciences, Patna, IND

**Keywords:** mutation, mycobacterium tuberculosis, drug resistance, gyrb, gyra, mdr-tb

## Abstract

Introduction: Multi-drug-resistant tuberculosis (MDR-TB) has become a major public health concern globally. Mutations in first- and second-line drug targets such as katG, inhA, rpoB, rrs, eis, gyrA, and gyrB have been associated with drug resistance. Monitoring predominant mutations in the MDR-TB patient population is essential to monitor and devise future therapeutic regimes. The present study is aimed to characterize genetic mutations in MDR isolates of *Mycobacterium tuberculosis *(MTB) bacilli conferring resistance to a second-line anti-tuberculosis drug in the Eastern Indian population.

Methods: This cross-sectional study was conducted in the Department of Microbiology, Indira Gandhi Institute of Medical Sciences, Patna, Bihar, and in the Tuberculosis Demonstration & Training Centre, Agamkuan, Patna. A total of 3270 patients suspected to have MDR-TB were recruited in the study. Two sputum samples, one on the spot, and the other in the morning were collected from each patient and the diagnosis of rifampicin-sensitive (RS)/rifampicin-resistant (RR/MDR) TB was done by Gene-Xpert test. One hundred fifty RS-TB samples and 150 RR/MDR-TB samples were considered for line probe assay (LPA). RS samples were subjected to first-line LPA using Genotype^®^ MTBDR Plus ver 2.0 and RR/MDR samples were considered for second-line LPA using Genotype^® ^MTBDRsl ver 2.0. All sputum samples were subjected to sputum smear microscopy using the Ziehl-Neelsen staining method. Statistical analysis was done using Statistical Package for Social Sciences (SPSS) version 26.0 (IBM Corp. Armonk, NY) and R (version 4.1; R Core Team 2021).

Results: In the present study, out of 3270 patients, we detected RR/MDR-TB in 235 patients (7.19%), RS-TB in 812 patients (24.83%), the rest of the patients negative for MTB (2223, 67.98%). Out of 150 RR/MDR-TB sputum samples tested, resistance to fluoroquinolone (FQ) was observed in 41 samples. The selected patients had predominantly FQ resistance due to the gyrA gene mutations (97.56%, n=40) compared to the gyrB gene mutations (2.44%, n=1). We observed >60% of the mutations in the gyrA gene in codon 94 (MUT3C (D94G), MUT3A (D94A), and MUT3D (D94H). In addition, we found the mutations MUT1 (A90V) and MUT2 (S91P) in the codons 90 and 91 of the gyrA gene in the considered MTB patient population.

Conclusion: The identified genes can be further validated to be considered as therapeutic targets, but more therapeutics and advanced strategies should be applied in the management of MTB.

## Introduction

The global burden of tuberculosis is among the major causes of human death. Multiple factors are responsible for compounding the disease burden that includes the social and economic context, hygiene, health management, access to treatment, and public awareness about the disease. However, the increasing death toll (1.6 million in 2021) remains a major concern. The estimated access to treatment for multi-drug-resistant tuberculosis (MDR-TB) patients is only one-third of the total patients [1].

The present standard treatment regimens remain effective, however, drug resistance, specifically MDR has become a serious hindrance to device treatment strategy and reduces the death toll for TB patients. The general TB treatment consists of first-line and second-line anti-tubercular drugs that target different gene products and hinders the pathogen’s growth and life cycle [2].

However, mutations in specific genes such as gyrA and gyrB in the case of fluoroquinolone (FQ) resistance, aid in developing the resistance in the pathogen and failing the treatment strategy. The large population of TB patients all over the world and in India have MDR-TB that requires immediate attention [3, 4].

In different countries, including India, monitoring and screening of the MDR-TB patient populations are regularly conducted to identify and monitor the dominant genetic mutations concerning common drugs such as rifamycin/rifampin (RIF), isoniazid (INH), pyrazinamide (PZA), ethambutol (EMB), FQ, etc [2, 5].

Therefore, population monitoring for the specific mutations in MDR-TB and novel treatment strategy devising are two important pillars to manage the burden of MDR-TB. Moreover, the growing understanding of the mutation spectrum and disease dynamics from regular monitoring of the mutations in TB patients may help in developing a new strategy in anti-tubercular therapy.

The present cross-sectional study was designed to understand the mutational pattern of genes among pulmonary MDR-TB strains and to identify the significant mutation types concerning the MDR and resistance type considered.

## Materials and methods

Study design and study population

This cross-sectional, 18-month long (March 2017 to August 2018) study was conducted in the Department of Microbiology, Indira Gandhi Institute of Medical Sciences, Patna, Bihar, and in Tuberculosis Demonstration & Training Centre, Agamkuan, Patna, an Intermediate Reference Laboratory (IRL) certified by Revised National Tuberculosis Control Program (RNTCP), Government of India. A total of 3270 patients suspected to have MDR-TB were recruited in the study. All patients suspected to have MDR-TB who visited these centers between March 2017 to August 2018 were recruited in the study. One hundred fifty RS-TB cases and 150 RR/MDR-TB cases were subjected to LPA for mutational analysis. 

Selection criteria and ethics consideration

The patients suspected of having multi-drug-resistant pulmonary tuberculosis (MDR-PTB) were included in this study; moreover, the MDR-TB suspect criteria used in the study were according to RNTCP guidelines. The inclusion criteria considered were failures of new TB cases (cases which were previously treated for TB and were smear positive even after completion of the most recent course of an anti-tubercular treatment regimen), positive smear results for previously treated cases that remain smear-positive at the fourth month onwards, all pulmonary TB cases that were contacts of known MDR-TB cases, all smear-positive cases previously treated pulmonary TB cases at diagnosis, any smear-positive follow-up result in new or previously treated cases, all smear-negative previously treated pulmonary TB cases at diagnosis, and HIV TB co-infected cases at diagnosis. Conversely, the exclusion criteria considered were the cases of extra-pulmonary TB and the cases of pulmonary TB that were below 14 years of age as cases below 14 years of age were considered to be pediatric cases.

The study protocols were approved by the Institutional Ethical Committee of Indira Gandhi Institute of Medical Sciences, Patna with approval number Memo No. 44/Acad dated 16.01.2017.

Sample collection

The demographic information and detailed clinical history of the patients were recorded along with the respective written informed consent. From each study participant, 3-5 ml of sputum was collected. Two sputum samples, one at the spot and the other in the morning were collected from each patient and were stored properly. The first specimen was collected during the first visit to the outpatient department, and the second sample was collected the next day early morning. Standard RNTCP protocol was followed for sample collection, storage, and transport. The collected samples were processed on the same day of collection or the next day. The samples that were processed on the next day were stored at 4°C-8°C.

Sample analysis

The sputum samples were collected in the morning from 150 patients of each category who were diagnosed with rifampicin-sensitive (RS)/rifampicin-resistant (RR/MDR) TB by Gene-Xpert test. This test is a rapid diagnostic test for tuberculosis detection as well as rifampicin resistance in direct smear-negative cases and requires minimal technical training. These samples were considered for LPA. RS samples were subjected to first-line LPA using Genotype® MTBDR Plus ver 2.0 and RR/MDR samples were considered for second-line LPA using Genotype®MTBDRsl ver 2.0 (Hain Lifesciences, Nehren, Germany). All sputum samples were subjected to sputum smear microscopy using the Ziehl-Neelsen staining method as per RNTCP guidelines.

Statistical analysis

All analyses were done using Statistical Package for Social Sciences (SPSS) version 26.0 (IBM Corp. Armonk, NY) and R (version 4.1; R Core Team 2021). Descriptive analyses were done for the collected data and the numerical results are presented as mean ± SD, and the categorical data are presented as percentages. After the detection of the specific mutations through experimentation, the absence (A) or presence (P) of the specific mutations was subjected to statistical significance analysis. Analyses were done for the significance of the mutation types concerning MDR type and resistance type. A contingency table was developed for each case and Chi-Square (χ2) test with continuity correction was conducted to understand the statistical significance of the respective case. P values less than 0.05 were considered to be significant.

## Results

Sample collection and assessment

Following the study protocol, sputum samples were collected from 3270 patients suspected to have MDR-PTB). RS-TB was detected in 24.83% (812/3270) of sputum samples and RR/MDR-TB was detected in 7.19% (235/3270) samples. Out of the total diagnosed cases of TB, RR/MDR-TB was detected in 22.44% (235/1047) of the samples, whereas 67.98% of the samples were found negative for TB. Further, molecular experiments were conducted on a total of 300 confirmed patients.

Patient demography

Out of the selected 300 patients, the majority of the patients (>70%), were in the age group of 15-45 years with a mean age of 40±16 years. Profiling of the disease occurrence in the patients of different age groups suggested that 40.67% of the MDR-TB and 44.66% of the RS-TB patients were within the age group of 31-45 years. This was followed by the age group 15-30 years with 30.67% of the MDR-TB patients and 25.33% of the RS-TB patients.

The analyses of the gender prevalence suggested that 69.33% of MDR-TB patients and 72% of the RS-TB patients were males, out of a total of 300 patients. Moreover, of the total patient population, 78.67% of the patients were from a rural region. Among the MDR-TB patients analyzed, 77.3% took previous anti-tubercular therapy for at least a month.

Clinical presentations

All the patients (n=300) were thoroughly examined by the designated physician for various clinical presentations. Most of the patients had cough for more than 2 weeks (82%), and persistent fever (55.33%). The other observed symptoms were loss of appetite, weight loss, hemoptysis, discomfort in the chest, and breathlessness (Table 1). 

**Table 1:** Clinical presentation of the patients (n=300)

Symptoms and signs	Frequency	Percent
Cough ≥ 2 weeks	246	82
Fever	16	55.33
Loss of appetite	110	36.66
Weight loss	88	29.33
Hemoptysis	53	17.66
Chest discomfort	96	32
Breathlessness	82	27.33

Microscopic analysis and line probe assay

Microscopic examinations and grading of the MDR-TB samples (n=150) suggested that most of the samples were of 2+ grade (37.33%, n=56), followed by 1+ grade (34.67%, n=52), and 3+ grade (28%, n=42). The samples were segregated into RR/MDR-TB, pre-XDR-TB, and XDR-TB based on the drug susceptibility evaluation. The Pre-XDR-TB and XDR-TB were detected in 25.33% and 5.33% (n=150) of the sputum samples, respectively. Further, 69.34 % of the samples were found to be susceptible to FQ and second-line injectable drugs (SLID). FQ resistance contributed to 86.84% (33/38) of pre-XDR-TB cases, while resistance to SLID was observed in 13.16% (5/38) of pre-XDR-TB cases.

Gene mutations and resistance strain analysis

FQ Resistance

Out of 150 sputum samples, 41 samples were FQ resistant. Moreover, Genotype® MTBDRsl ver 2.0 suggested mutation in the gyrA gene (97.56%, n=41) and gyrB gene (2.44%, n=41) for FQ-resistant samples. The majority of the mutations in the gyrA gene (60.97%, n=41) were observed at codon 94. Other gyrA mutations were detected at codon 90 (36.60%, n=41) and codon (91, n=2.43%), respectively.

The following types of single-defined mutations were detected at gyrA codon 94: gyrA MUT3C (D94G) (18/41, 43.9 %), gyrA MUT3A (D94A) (4/41, 9.76%), gyrA MUT3D (D94H) (1/ 41, 2.43%), and gyrA MUT3B (D94N/D94Y) (2/41, 4.87%). Other gyrA-defined mutations detected at codons 90 and 91 were: gyrA MUT1 (A90V) (15/41, 36.60%) and gyrA MUT2 (S91P) (1/ 41, 2.43%). The observed hybridization patterns were noted for gyrA gene mutations (Table 2).

**Table 2:** Hybridization patterns observed in FQ-resistant strains for the gyrA gene + = presence of mutation, Δ = absence of mutation.

Hybridization band(s) observed	Mutation(s) detected	Frequency of mutations	Percentage
ΔWT1	None	1	2.44
ΔWT1 &/or ΔWT2	None	0	0
ΔWT, MUT1+	A90V	14	3.41
ΔWT, MUT2+	S91P	1	2.44
ΔWT, MUT3A+	D94A	4	9.76
ΔWT, MUT3B+	D94N/Y	2	4.87
ΔWT, MUT3C+	D94G	17	41.46
ΔWT, MUT3D+	D94H	1	2.44
WT+, MUT1+	A90V	1	2.44

In the case of the gyrB gene, WT1 (536-541) probe was missing in one of the strains, and no substitution in the gyrB gene was observed.

Second-Line Injectable Drug (SLID) Resistance

Obtained results suggested that 13 samples were resistant to SLID. We observed a total of 12 mutations in the rrs gene and one mutation in the eis gene. In the rrs gene, all the mutations were observed at MUT1 (A1401G). In the eis gene, hybridization was observed only at probe MUT1(C-14T) with the loss of wild-type probe WT2 (14,12,10). The distribution of mutation types suggested the prevalence of ΔWT, MUT1+(69.2%) hybridization pattern in the rrs gene (Table 3).

**Table 3:** Hybridization patterns observed in strains resistant to SLID + = presence of mutation, Δ = absence of mutation

Gene	Hybridization band(s)	Mutation(s)	Frequency	Percentage
RRS	WT+, MUT1+	A1401G	3	23.1
RRS	ΔWT, MUT1+	A1401G	9	69.2
EIS	ΔWT2, MUT1+	C-14T	1	7.7
Total			13	100

Even though less, XDR-TB was detected in 5.33% (n=150) samples. It was observed that the mutations in the gyrA gene related to FQ resistance were associated with the absence of wild-type probes and the development of hybridization at codon 94 (MUT3A, MUT3B, MUT3C, and MUT3D), and at codon 90 (MUT1) in 75% and 25% samples, respectively. Probes gyrAWT3 and gyrAWT2 were missing in 75% (n=8) and 25% of cases, respectively. The absence of probes gyrA WT2 and gyrAWT3 was associated with the development of hybridization at codon 90 (MUT1) and 91 (MUT2), respectively. Similarly, the LPA for the first line was also conducted and isoniazid-related resistance of the strains was evaluated and the mutations were analyzed for the inhA gene and katG gene (data not shown).

Outcomes of the Mutations in Different MDR Types

All observed mutation data for MDR was analyzed based on the presence (P) or absence (A) of the mutation for a particular gene. The mutations considered for this study related to MDR were for gyrA (WT1, WT2, WT3, MUT1, MUT2, MUT3A, MUT3B, MUT3C, and MUT3D), gyrB (WT1, WT1_18, WT2, MUT1, MUT2, MUT1_20, MUT2_21), and EIS (WT1, WT2, WT3, MUT1) genes (Table 4).

**Table 4:** Analysis outcomes of the mutations concerning the MDR types WT = wild type, MUT = mutations, MDR = multiple-drug resistance, XDR = extensive drug resistance, A = absent, P = present.

Contingency tables					
gyrA_WT1	MDR	pre-XDR	XDR	Total	P
A	0	1	0	1	0.227
P	104	37	8	149	
Total	104	38	8	150	
gyrA_WT2	MDR	pre-XDR	XDR	Total	
A	0	14	1	15	
P	104	24	7	135	
Total	104	38	8	150	
gyrA_WT3	MDR	pre-XDR	XDR	Total	
A	0	18	7	25	
P	104	20	1	125	
Total	104	38	8	150	
gyrA_MUT1	MDR	pre-XDR	XDR	Total	
A	104	24	7	135	
P	0	14	1	15	
Total	104	38	8	150	
gyrA_MUT2	MDR	pre-XDR	XDR	Total	
A	104	37	8	149	0.227
P	0	1	0	1	
Total	104	38	8	150	
gyrA_MUT3A	MDR	pre-XDR	XDR	Total	
A	104	35	6	145	
P	0	3	2	5	
Total	104	38	8	150	
gyrA_MUT3B	MDR	pre-XDR	XDR	Total	
A	104	38	5	147	
P	0	0	3	3	
Total	104	38	8	150	
gyrA_MUT3C	MDR	pre-XDR	XDR	Total	
A	104	24	6	134	
P	0	14	2	16	
Total	104	38	8	150	
gyrA_MUT3D	MDR	pre-XDR	XDR	Total	
A	104	37	8	149	0.227
P	0	1	0	1	
Total	104	38	8	150	
gyrB_WT1	MDR	pre-XDR	XDR	Total	
A	0	2	0	2	0.05
P	104	36	8	148	
Total	104	38	8	150	
gyrB_MUT1	MDR	pre-XDR	XDR	Total	
A	104	38	8	150	
Total	104	38	8	150	
gyrB_MUT2	MDR	pre-XDR	XDR	Total	
A	104	38	8	150	
Total	104	38	8	150	
gyrB_WT1_18	MDR	pre-XDR	XDR	Total	
A	0	5	6	11	
P	104	33	2	139	
Total	104	38	8	150	
gyrB_WT2	MDR	pre-XDR	XDR	Total	
P	104	38	8	150	
Total	104	38	8	150	
gyrB_MUT1_20	MDR	pre-XDR	XDR	Total	
A	104	33	0	137	
P	0	5	8	13	
Total	104	38	8	150	
gyrB_MUT2_21	MDR	pre-XDR	XDR	Total	
A	104	38	8	150	
Total	104	38	8	150	
eis_WT1	MDR	PRE-XDR	XDR	Total	
P	104	38	8	150	
Total	104	38	8	150	
eis_WT2	MDR	pre-XDR	XDR	Total	
A	0	1	0	1	0.227
P	104	37	8	149	
Total	104	38	8	150	
eis_WT3	MDR	pre-XDR	XDR	Total	
P	104	38	8	150	
Total	104	38	8	150	
eis_MUT1	MDR	pre-XDR	XDR	Total	
A	104	35	8	147	0.011
P	0	3	0	3	
Total	104	38	8	150	

Further, the significance of the mutations was analyzed based on resistance groups also, such as various combinations of fluoroquinolones (FQ), second-line treatment (SL), and second-line injectable drugs (SLID). The resistance group considered was for FQ, FQ+SLID, S, and SLID study groups for different genes with their respective wild type (WT) and mutated (MUT) variations (Table 5).

**Table 5:** Statistical analysis of the mutations for different resistance categories WT = wild type, MUT = mutations, FQ = fluoroquinolones, SLID = second-line injectable drugs, A = absent, P = present

Contingency tables						
gyrA_WT1	FQ	FQ+SLID	S	SLID	Total	P
A	1	0	0	0	1	0.294
P	31	8	104	6	149	
Total	32	8	104	6	150	
gyrA_WT2	FQ	FQ+SLID	S	SLID	Total	
A	14	1	0	0	15	
P	18	7	104	6	135	
Total	32	8	104	6	150	
gyrA_WT3	FQ	FQ+SLID	S	SLID	Total	
A	18	7	0	0	25	
P	14	1	104	6	125	
Total	32	8	104	6	150	
gyrA_MUT1	FQ	FQ+SLID	S	SLID	Total	
A	18	7	104	6	135	
P	14	1	0	0	15	
Total	32	8	104	6	150	
gyrA_MUT2	FQ	FQ+SLID	S	SLID	Total	
A	31	8	104	6	149	0.294
P	1	0	0	0	1	
Total	32	8	104	6	150	
gyrA_MUT3A	FQ	FQ+SLID	S	SLID	Total	
A	29	6	104	6	145	
P	3	2	0	0	5	
Total	32	8	104	6	150	
gyrA_MUT3B	FQ	FQ+SLID	S	SLID	Total	
A	32	5	104	6	147	
P	0	3	0	0	3	
Total	32	8	104	6	150	
gyrA_MUT3C	FQ	FQ+SLID	S	SLID	Total	
A	18	6	104	6	134	
P	14	2	0	0	16	
Total	32	8	104	6	150	
gyrA_MUT3D	FQ	FQ+SLID	S	SLID	Total	
A	31	8	104	6	149	0.294
P	1	0	0	0	1	
Total	32	8	104	6	150	
gyrB_WT1	FQ	FQ+SLID	S	SLID	Total	
A	2	0	0	0	2	0.058
P	30	8	104	6	148	
Total	32	8	104	6	150	
gyrB_MUT1	FQ	FQ+SLID	S	SLID	Total	
A	32	8	104	6	150	
Total	32	8	104	6	150	
gyrB_MUT2	FQ	FQ+SLID	S	SLID	Total	
A	32	8	104	6	150	
Total	32	8	104	6	150	
gyrB_WT1_18	FQ	FQ+SLID	S	SLID	Total	
A	0	6	0	5	11	
P	32	2	104	1	139	
Total	32	8	104	6	150	
gyrB_WT2	FQ	FQ+SLID	S	SLID	Total	
P	32	8	104	6	150	
Total	32	8	104	6	150	
gyrB_MUT1_20	FQ	FQ+SLID	S	SLID	Total	
A	32	0	104	1	137	
P	0	8	0	5	13	
Total	32	8	104	6	150	
gyrB_MUT2_21	FQ	FQ+SLID	S	SLID	Total	
A	32	8	104	6	150	
Total	32	8	104	6	150	
eis_WT1	FQ	FQ+SLID	S	SLID	Total	
P	32	8	104	6	150	
Total	32	8	104	6	150	
eis_WT2	FQ	FQ+SLID	S	SLID	Total	
A	0	0	0	1	1	
P	32	8	104	5	149	
Total	32	8	104	6	150	
eis_WT3	FQ	FQ+SLID	S	SLID	Total	
P	32	8	104	6	150	
Total	32	8	104	6	150	
eis_MUT1	FQ	FQ+SLID	S	SLID	Total	
A	32	8	104	3	147	
P	0	0	0	3	3	
Total	32	8	104	6	150	

The outcome of the analysis suggested that certain cases were statistically significant for the presence or absence of the mutation for MDR, pre-XDR, or XDR. In most of the cases, the presence or absence of mutation was found to be statistically significant for MDR, pre-XDR, or XDR types (P<0.001). For instance, the statistically significant contribution of mutations was found for gyrAWT2, gyrAWT3, gyrAMUT1, gyrAMUT3A, gyrAMUT3B, gyrAMUT3C, gyrBMUT1, gyrBMUT2, gyrBWT118, gyrBWT2, gyrBMUT120, gyrBMUT221, eisWT1, and eisWT3 (Table 4).

Similarly, contingency table development and Chi-Square (χ2) test were conducted for the presence or absence of mutations concerning various resistance groups considered for this study. It was observed that the presence or absence of mutations in various groups was statistically significant (P<0.001) for different resistance group considerations, except for the gyrAWT1, gyrAMUT2, gyrAMUT3D, and gyrBWT1 (Table 5). The analysis outcomes suggest that for the considered study population, most of the genetic mutations studied for the specific genes (gyrA, gyrB, eis) may have a significant role in the virulence or drug resistance capabilities of the pathogen.

However, we have also observed that mutations in some of the specific gene types, such as gyrAWT1, gyrAMUT2, gyrAMUT3D, gyrBWT1, eisWT2 in relation to MDR type, and gyrAWT1, gyrAMUT2, gyrAMUT3D, and gyrBWT1 concerning resistance type may not have any statistically significant contribution in the pathogen virulence or drug resistance for the considered study population.

## Discussion

Prevalence of TB is present all across the globe with a heavy death toll of 1.6 million patients in 2021 alone. Due to TB, the affected patient population was 10.6 million globally in 2021, the second leading infectious disease after COVID-19, and the thirteenth leading cause of death worldwide [1]. The scenario in India is not different, the survey suggested that two-thirds of the global disease burden is borne by eight countries among which India leads with a TB incidence burden of 27% [6]. Further, MDR remains a major public health concern, and the need for novel and effective drug development has become a challenge. Therefore, understanding the underlying cause of MDR will have a tremendous impact on the future decision of treatment regimens to tackle TB cases. In the present study, we analyzed patients suspected to have MDR-TB and observed that the majority of the MDR-TB patients had FQ resistance due to the gyrA gene mutations. The majority of the gyrA gene mutations were in the codon 94 (MUT3C (D94G), MUT3A (D94A), MUT3D (D94H)).

Globally, TB treatment is done through majorly first-line and second-line therapeutics. The first-line drugs (FLD) are predominantly rifamycin/rifampin (RIF), isoniazid (INH), pyrazinamide (PZA), ethambutol (EMB), etc [2]. On the other hand, the second line of drugs are different fluoroquinolones such as moxifloxacin (MOX), ofloxacin (OFX), and others. However, at molecular and socioeconomic levels there are disadvantages to using SLID such as more toxicity and less accessibility to the drugs, and more expense.

All these therapeutics, first-line or second-line, are targeted to specific gene expression or enzyme products [2]. For instance, INH is targeted to katG and inhA, related to catalase/peroxidase and enoyl reductase, respectively. Similarly, fluoroquinolones are targeted to the gene expression of gyrA/gyrB, i.e., DNA gyrase. Mutations of such targeting genes were found to be the major cause of drug resistance in TB [5, 7]. Moreover, understanding population-specific mutations may provide a better understanding of the MDR for a specific geographical region or a specific population. Jadaun et al. (2012) unveiled the specific role of embCAB gene mutations regarding ethambutol resistance in *Mycobacterium tuberculosis* (MTB) in the Indian region [8]. The recent evaluation of FQ-resistant TB patients in India suggested that >25% and >44% FQ resistance is present in the newly diagnosed and follow-up rifampicin-resistant TB (RR-TB) patients, respectively. The genetic identification of the mutations further revealed that the MUT3C in the gyrA gene was predominant among the FQ-resistant patient samples [9].

Hence, the molecular analyses of the gene mutations may reveal the mechanism of MDR-TB in a specific population. Several earlier studies have attempted to understand the genetic mutations that are probable reasons for drug resistance in MTB [10]. A systematic review conducted by Georghiou et al. (2012) suggested that kanamycin (KAN), amikacin (AMK), and capreomycin (CAP) resistance predominantly occurred due to rrs A1401G mutation which was also detected in 7% of CAP susceptible TB strains. The complexity of resistance of global strains was unveiled when additional mutations such as rrs, eis, and others were found associated with the resistant strains [11].

The screening of MTB strains is essential to understand the predominant mutant types [9]. Similar mutation analyses have been conducted in other countries and patient populations such as in African countries, China, the Middle East, the European region, and other countries including India [12-17].

Functional genetic analysis of the mutations for gyrA and gyrB genes in FQ-resistant TB revealed the possible mutational spectrum related to the quinolone resistance determining region (QRDR) region of gyrA, and other genetic regions for these genes that are responsible for the drug resistance [18]. Multiple genetic mutations in gyrA and gyrB genes and their significant association and relevance with clinically proven FQ resistance have been studied [18]. Such analyses of the specific mutations in gyrA and gyrB genes and their association with FQ resistance were studied for specific patient populations in other countries and in India [19-21]. In the Chinese population, analysis of the QRDR in gyrA in clinical isolates of levofloxacin-resistant MTB showed single codon mutations of H70R, A90V, S91A, D94G, D94A, or D94N, and double codon mutation of A90V with D94A [19]. A similar study conducted on Indian TB patients in the New Delhi region suggested the presence of 27 mutations in the gyrA genes analyzed from 25 strains MTB strains, the predominant mutation observed was D94G [21].

In the present study, we observed the RR/MDR-TB in 7.19% (n=3270) and RS-TB in 24.83% (n=3270) of the patients screened. Predominant FQ resistance was observed due to the gyrA gene mutations compared to the gyrB gene mutations. In consistency with other reports, we have observed that over 60% of the mutations in the gyrA gene were in codon 94, followed by codon 90 (37%) and 91 (3%). The observed specific single codon mutations in gyrA codon 94 were MUT3C (D94G), MUT3A (D94A), MUT3D (D94H), and MUT3B (D94N/D94Y). In codons 90 and 91, the observed mutations were gyrA MUT1 (A90V) and gyrA MUT2 (S91P) for the considered MTB patient population.

Limitations

The study was conducted in a tertiary care center. Therefore, the findings can not be generalized to the general population of patients. Single center nature of the study also limits the conclusions presented. The mutation profiles observed in the study should be further validated by high-throughput genetic sequencing techniques such as next-generation sequencing (NGS). Also, phylogenetic analysis of the observed mutations was not conducted. Therefore, further multi-centric and comprehensive studies employing high throughput genetic approaches should be conducted to validate the findings of the present study.

## Conclusions

In the present study, we have reported the specific predominant and statistically significant mutations observed in gyrA and gyrB genes responsible for fluoroquinolone resistance. However, apart from monitoring the specific mutations in the population, novel therapeutics and advanced strategies should be defined to overcome the problem of drug resistance in tuberculosis treatment. 
